# Overexpression of S6 Kinase 1 in Brain Tumours Is Associated with Induction of Hypoxia-Responsive Genes and Predicts Patients' Survival

**DOI:** 10.1155/2012/416927

**Published:** 2012-04-05

**Authors:** Heba M. S. Ismail

**Affiliations:** Cancer Biology Department, National Cancer Institute, Cairo University, Cairo 11796, Egypt

## Abstract

mTOR/S6K pathway is a crucial regulator of cell growth and metabolism. Deregulated signalling via S6K has been linked to various human pathologies, including metabolic disorders and cancer. Many of the molecules signalling upstream of S6K have been shown to be either mutated or overexpressed in tumours, leading to S6K activation. The role of S6K1 in brain tumours is not fully investigated. In this study, we investigated the gene expression profile of S6 kinases in brain and CNS tumours using the publically available Cancer Microarray Database. We found that S6K1 but not S6K2 gene is overexpressed in brain tumours and this upregulation is associated with patients' poor survival. Furthermore, we interrogated Oncomine database for the expression profile of hypoxia-induced genes using a literature-defined concept. This gene list included HIF1A, VEGFA, SOX4, SOX9, MMP2, and NEDD9. We show that those genes are upregulated in all brain tumour studies investigated. Additionally, we analysed the coexpression profile of S6K1 and hypoxia responsive genes. The analysis was done across 4 different brain studies and showed that S6K1 is co-overexpressed with several hypoxia responsive genes. This study highlights the possible role of S6K1 in brain tumour progression and prediction of patients' survival. However, new epidemiological studies should be conducted in order to confirm these associations and to refine the role of S6K1 in brain tumours as a useful marker for patients' survival.

## 1. Introduction

Brain and other central nervous system (CNS) cancers include a variety of histopathologic subtypes, but the most common, by far, are gliomas. These tumours, which arise from the glial cells that surround and support neurons, include astrocytoma, glioblastoma, oligodendroglioma, oligoastrocytoma, and ependymoma. Medulloblastoma, another neuroepithelial cancer, is relatively common in children but rare in adults. Brain cancers in children typically arise in the cerebellum, whereas brain cancers in adults are more likely to occur in the cerebral hemispheres [1]. In adults, older age at diagnosis of brain cancer is associated with higher tumour grade and poorer prognosis. Indeed, glioblastoma is among the most lethal of all cancers. Brain and central nervous system (CNS) tumours occur at each stage of life and are therefore classified as embryonic, paediatric, and adult cancers [2, 3]. 

According to Central Brain Tumour Registry of the Unites States (CBTRUS), the prevalence rate for all primary brain and central nervous system tumours was estimated to be 209.0 per 100,000 in 2004 [4]. The five-year relative survival rate following diagnosis of a primary malignant brain and central nervous system tumour is 33.8% for males and 37.5% for females (1995–2007 data) [5]. In Egypt, brain and other CNS cancers accounted for 3.1% of all cancers in Egyptians, a large majority of cancers were located in the brain (85.2%) (Middle East Cancer Consortium 1995–2001) [6].

 Due to the lack of effective therapies for aggressive brain and CNS tumours, the identification of new targets and prognostic indicators is required. Current studies in this area are focused on developing new therapies that target specific molecular events that lead to malignant transformation of cells [7]. 

The PI3K/Akt pathway is one of the major cell survival pathways activated on stimulation of receptor tyrosine kinases such as epidermal growth factor receptors (EGFR) that are over expressed in 40–60% of gliomas [8–10]. Activation of PI3K/Akt pathway has been associated with malignant transformation of cells and is frequently overexpressed in glioblastoma tumours when compared to nonglioblastoma tumours [11]. This activation is also associated with increased tumour grade that correlates positively with adverse clinical outcome in gliomas [12]. Mammalian target of rapamycin (mTOR) is a serine/threonine kinase that functions downstream of the PI3K/Akt pathway [13]. mTOR is known to regulate cell proliferation, growth, and survival by regulating translation initiation. Akt is shown to activate mTOR through inhibition of TSC1/2 (tuberous sclerosis complex 1 and 2) and activation of Ras homologue-enriched in brain (Rheb) [14]. Upon activation by mTOR, S6K1 phosphorylates S6 ribosomal protein, leading to increased translation of mRNA with oligopyrimidine tract at the 5′ terminal (5′TOP) [15]. 

S6K1 itself has no specific inhibitors that are available commercially but it responds to inhibitors that target its upstream regulators as mTOR and PI3K. Rapamycin (sirolimus), a macrolide antibiotic, blocks mTOR kinase activity by forming a complex with FK506-binding protein (FKBP-12), thereby leading to the blockade of translation initiation through its action on S6K and 4EBP1 and cell cycle arrest at G1 phase [16, 17]. Rapamycin's growth inhibitory action has also been correlated with a decrease in glucose and amino acids uptake by rapamycin-sensitive glioblastoma cells [18]. Several clinical trials of rapamycin and its derivatives are being conducted to evaluate their efficacy [19]. Rapamycin and its derivatives have been shown to inhibit growth in several cancers, including breast cancer, pancreatic cancer, prostate cancer, melanoma, renal cell cancer, leukemia, and glioblastoma [20–22]. Phase II trial with temsirolimus, an ester analog of rapamycin, showed that this drug was well tolerated in patients with recurrent glioblastoma and this study has also shown that patients with high baseline levels of S6K1 responded to the drug treatment [23].

Using human glioma cell lines and transformed human astrocytes, Nakamura et al., 2008, have found that suppression of mTOR or raptor was sufficient to significantly reduce anchorage-independent growth in soft agar, an assay of transformation. Furthermore, S6K1, but not eIF4E, rescued glioma growth in soft agar from rapamycin-mediated suppression, and transient S6K1 inhibition was sufficient to significantly reduce glioma growth in soft agar. Additionally, they found that *in vivo *S6K1 suppression in intracranially implanted glioma xenografts reduced levels of phosphorylated S6 and also resulted in reduced intracranial tumour growth. Their findings define a significant role for the mTOR-raptor (mTORC1)-S6K pathway in supporting gliomagenesis [24].

The ribosomal protein S6 kinase (S6K) family is a key regulator of cell growth and cell size and acts downstream of the PI3K/mTOR pathway, and it has been identified to date to exert crucial functions in many other cellular processes such as metabolism and transcription regulation. The importance of the PI3K and mTOR pathways in the regulation of S6K activity was revealed by the ability of inhibitors of those pathways to block the activity of S6 kinases. Activating mutations in kinases involved in signalling via the PI3K/mTOR/S6K pathway were identified in many tumours indicating the oncogenic nature of this signalling pathway. On the other hand, inhibitory mechanisms were described to target S6K pathway through inhibiting its upstream signalling. Inhibitory mechanisms are exerted by a number of tumour suppressor genes such as PTEN, TSC1/2, REDD1, and others. Deregulation of those proteins in many tumours has resulted in activation of PI3K and mTOR pathways and in turn to S6K activation. This has raised the possibility that S6 kinases are involved in tumour progression processes (reviewed in [25]).

The initial link between mTOR/S6K regulation and the cellular response to hypoxia has come from several studies, which have demonstrated that mTOR signalling upregulates expression of the hypoxia inducible factor (HIF-1*α*) and vascular endothelial growth factor (VEGF), reviewed in [30].

Recently, a TOS motif has been found in HIF-1*α* subunit, which also binds to raptor [31]. Loss of the TOS motif disrupts regulation of hypoxia via HIF-1*α*. Immunofluorescent analysis of S6K1 and AKT phosphorylation status has revealed positive correlation between a high p-Akt and p-S6K expression, and a venous and capsular invasion of hepatocellular carcinoma (HCC) [32]. This suggests that activation of the Akt-mTOR-S6K pathway plays a significant role in HCC progression by promoting neoangiogenesis.

A proposed model for the inhibition of mTOR/S6K pathway occurs under hypoxic conditions through coordination of different upstream tumour suppressors. This model involves three different mechanisms: (1) REDD1 upregulation, (2) AMPK activation resulting in mTOR inhibition through the activation of the TSC1/TSC2 tumour suppressor complex [33, 34], and (3) mTOR accumulation in the nucleus through the action of the PML tumour suppressor [35]. All three regulatory events inhibit the interaction of mTOR with Rheb, which in turn reduces the phosphorylation/activation of S6K. Inhibition of the mTOR/S6K pathway through these mechanisms results in decreased translation of HIF-1*α* and expression of VEGF [33–35]. Deregulation of these tumour suppressors in cancer results in activation of the mTOR/S6K pathway and subsequent elevation of HIF-1*α* and VEGF, which promote tumour angiogenesis. The combination of mTOR inhibitors with angiogenesis inhibitors could be a useful tool for cancer therapy.

In this study, we aimed to investigate the gene expression profile of S6 kinases in human brain tumours and its association with patients' clinical outcomes as response to treatment, recurrence, and survival. Additionally, we performed coexpression analysis of S6 kinases and hypoxia-induced genes. We performed those analysis using publically available cancer microarray database; ONCOMINE.

## 2. Materials and Methods

### 2.1. Databases

We used Oncomine Cancer Microarray database (http://www.oncomine.org/) [26] to study the profile of S6K1, S6K2, and hypoxia-induced genes expression in human brain and CNS tumour types versus their normal tissue counterparts. In order to compare the gene expression in a tumour type to its normal counterpart, gene expression data from a same study, performed with the same methodology, were used. The gene expression data were log transformed, median centered per array, and the standard deviation was normalized to one per array [26]. A gene was considered as overexpressed when its mean value in tumour samples was significantly higher to its mean value in the normal tissue counterpart using a *t*-test (*P* ≤ 0.05) and the fold of induction was ≥1.5. Brain and CNS cancer data sets used in this study are summarised in Table 1.

### 2.2. Statistical Analysis

The results were analysed using GraphPad prism computer system (GraphPad Software, San Diego, USA). Statistical analysis comparisons were done with Mann-Whitney or student *t*-tests for gene expression analysis. 

## 3. Results

### 3.1. S6 Kinase 1 Is Overexpressed in Human Brain Tumours

**Figure 1:**
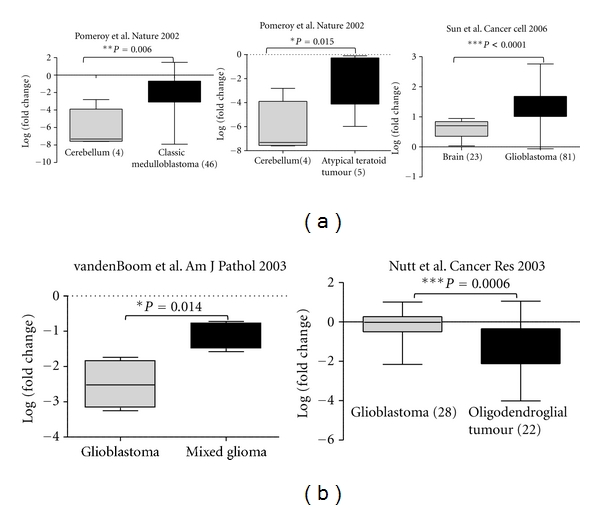
S6K1 is overexpressed in brain tumours in Oncomine database. Data sets in a single panel were from the same study. Gene expression profile of S6K1 gene (GEP) data are log transformed and normalized as previously described [26]. In brackets, are indicated the number of patients in each category. (a) Comparison between S6K1 gene expression in brain tumours against normal counterparts. (b) Comparison between S6K1 gene expression in different histopathological types of brain tumours.

In the present study, we queried the Oncomine database to systematically assess relative gene expression levels of S6K1 and S6K2 genes in brain and CNS tumours. Gene expression data from embryonic, pediatric, and adult brain tumours were collected from Oncomine database (http://www.oncomine.com/) [26]. We compared gene expression in normal brain versus cancer tissues, and in different histological subtypes. Differential analysis of S6 kinases gene expression in brain and CNS tumours versus normal tissue counterparts has retrieved 9 results in Oncomine database. We have analysed the studies that showed a significant difference value of gene expression (*P* ≤ 0.05) in cancerous tissues compared with normal counterparts. S6K1 gene (RPS6KB1) is significantly overexpressed in different brain tumours compared with normal brain tissue in 4 independent studies. On the other hand, S6K2 (RPS6KB2) gene expression profile did not change between cancerous and normal brain tissues (data not shown). S6K1 gene expression is significantly upregulated in Pomeroy et al. study [28] that analysed four different tumour types against normal cerebellum; atypical teratoid/rhabdoid tumour (fold = 20.023, *P* = 0.015), classic medulloblastoma (fold = 13.827, *P* = 0.02), desmoplastic medulloblastoma (fold = 6.658, *P* = 0.05), and malignant glioma (fold = 5.389, *P* = 0.06). While in Sun et al. study [36], S6K1 is overexpressed in glioblastoma compared to normal brain (fold = 1.57, *P* = 4.21*E* − 11), and in anaplastic astrocytoma (fold = 1.5, *P* = 4.72*E* − 5). Furthermore, we have found that S6K1 is overexpressed in glioblastoma compared to normal brain in a Murat brain study, 2008 [43] (fold = 1.644, *P* = 9.89*E* − 4).  Figure 1(a) shows some representative results of the analysis performed of S6K1 in brain tumours against normal brain. 

Furthermore, we have found that S6K1 is differentially overexpressed in specific histology types of brain tumours. In van den Boom et al. study [42], S6K1 gene is differentially overexpressed in mixed glioma when compared to astrocytoma and glioblastoma (*P* = 0.017). While in Nutt et al. study [29], S6K1 is differentially overexpressed in glioblastoma against oligodendroglial tumour (*P* = 0.002). (Figure 1(b)).

These results indicate that S6K1 gene but not S6K2 is overexpressed in a number of brain and CNS tumours and shows differential expression profile in specific histological types. 

### 3.2. S6K1 Overexpression Is Associated with Patients' Poor Survival

**Figure 2:**
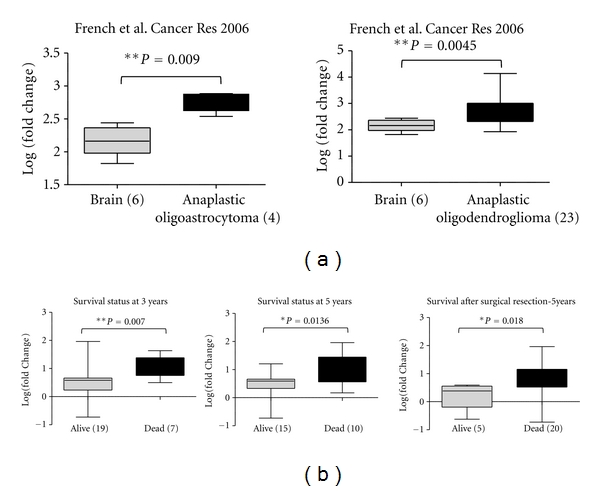
S6K1 gene overexpression in brain tumours compared to normal brain is associated with patients' poor survival. Data sets in a single panel were from the same study. Gene expression profile [GEP] data are log transformed and normalized as previously described [26]. In brackets, are indicated the number of samples. (a) Comparison of S6K1 gene expression profile in brain tumours French Brain Study [27] compared to normal brain. (b) Comparison of S6K1 gene expression profile in brain tumours in both alive and dead subjects at 3 and 5 years of survival.

To assess the clinical significance of S6K1 overexpression in brain tumours, we investigated the association between its gene expression levels and the patient's clinical outcome including response to treatment, recurrence, and survival status. No significant association between S6K1 gene expression levels and patients response to therapy or sample recurrence status was identified, while a significant association between S6K1 overexpression and patients' poor survival status was observed. In French et al. study [27], S6K1 gene is significantly overexpressed in anaplastic oligoastrocytoma compared to normal brain (*P* = 5.20*E* − 4) and in anaplastic oligodendroglioma compared to normal brain (*P* = 0.0045, Figure 2(a)). In the same study, as shown in Figure 2(b), S6K1 is significantly overexpressed in patients who died later after 3 and 5 years of diagnosis (*P* = 0.007 and *P* = 0.018, resp.). Additionally, we found that S6K1 is overexpressed in patients who died after 5 years of surgical resection compared to alive patients (*P* = 0.0136) [27]. Furthermore, we have tested the association between S6K1 gene expression and patients' survival by analysing the data available from Pomeroy et al. study [28]. We have found that, in classic medulloblastoma, S6K1 overexpression is significantly associated with patients' poor survival (*P* = 0.04) (supplementary Figure  1(a) available at doi:10.1155/2012/416927).

Taken together, we conclude that S6K1 is overexpressed in patients that showed poor survival status. This indicates that S6K1 gene expression could be used as a useful marker to predict patients' survival.

### 3.3. Hypoxia Induced Genes Are Overexpressed in Human Brain Tumours

The adaptation of tumours to hypoxia is critical for their survival and growth. The high proliferation rate of solid tumours causes the continuous outstripping of the oxygen supply provided by the local vasculature, resulting in hypoxic regions within the tumour. Hypoxia inducible factor (HIF) is the key mediator of cellular response to hypoxia, activating the expression of multiple genes that participate in angiogenesis, iron metabolism, glycolysis, glucose transport, and cell proliferation and survival. To assess the gene expression profile of hypoxia-induced genes in brain and CNS tumours, we queried the oncomine database for the following genes; HIF1A1, VEGFA, MMP2, NEDD9, SOX4, and SOX9. Those genes were selected based on literature. We found that hypoxia-induced genes are overexpressed in a number of brain tumours.

Table 2 summarizes the analysis results of the selected genes indicating the induction fold and the significance difference of gene expression in a tumour type compare to normal brain. Only analysis that showed fold of induction >2 and *P* < 0.01 was included. The results we show here highlights the importance of hypoxic mechanism in brain tumours and the high level of overexpression of angiogenic factors as HIF1A, VEGFA, and MMP2 that are implicated in metastasis could be target molecules in those tumour types.

### 3.4. S6K1 Is Coexpressed with Genes Induced by Hypoxia

**Figure 3:**
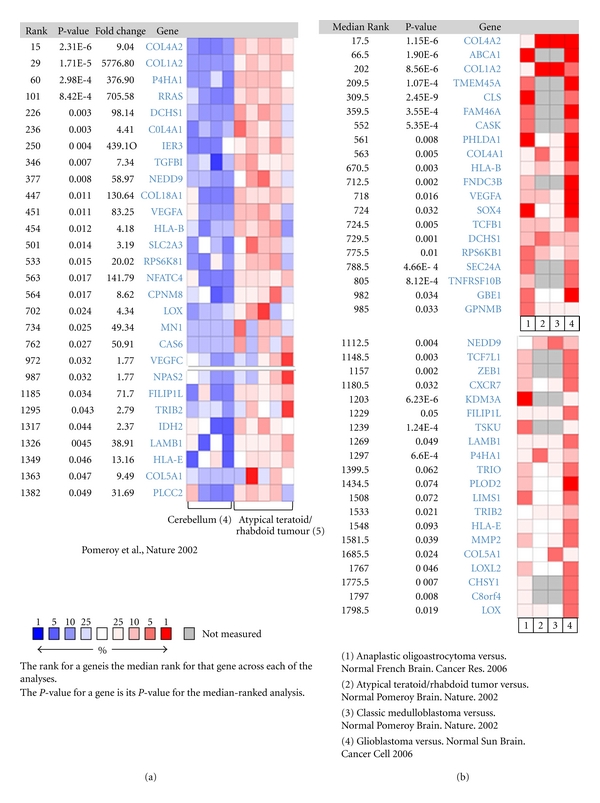
S6K1 gene is coexpressed with hypoxia responsive genes in oncomine database. (a) Heat map of coexpression profile of S6K1 and hypoxia induced genes in atypical teratoid tumours compared with normal cerebellum Pomeroy brain study [28]. (b) Comparison of co-expression gene profile of S6K1 and hypoxia induced genes across 4 different analyses [27–29].

S6K1 regulates cell size and metabolism. Only recently, a connection between mTOR/S6K pathways to hypoxia was suggested by different studies. Here, we aimed to investigate the coexpression profile of S6K1 and hypoxia-induced genes in brain tumours. To achieve that, we queried the oncomine database using a literature defined concept “Concept: Upregulated genes in response to hypoxia and in response to HIF-1 expression.” This concept included a gene list of genes reported in literature to be upregulated by hypoxia and in response to hypoxia inducible factor 1. This gene list incorporated HIF1A, VEGFA, SOX4, SOX9, NEDD9, CLO4A1, COL1A1, and other genes. The query included S6K1 has been done in brain and CNS tumours versus normal brain. We have observed that S6K1 is co-overexpressed with known genes that are induced by hypoxia.  Figure 3(a) shows a heat map of coexpression profile analysis of S6K1 and hypoxia-induced genes in Pomeroy brain study [28]. The analysis showed a significant overexpression of S6K1 and hypoxia-induced genes in atypical Teratoid/Rhabdoid Tumour compared to normal cerebellum (Figure 3(a)). To extend the analysis to more studies we compared the expression profile of S6K1 and hypoxia-induced genes in 4 different brain tumours comprising 3 independent studies [27–29] (298 patients).  Figure 3(b) shows the comparison of S6K1 and hypoxia-induced genes expression profile across 4 different analyses. We noticed that VEGFA, HIF1A, SOX4, SOX9, MMP2, TGFB1 genes are overexpressed together with S6K1 in all 4 tumour types investigated.

Taken those results together, we indicate that S6K1 overexpression in brain tumours is associated with overexpression of hypoxia-induced genes and reflects a possible connection between S6K1 and hypoxia in that tumour type.

## 4. Discussion

mTOR/S6K pathway plays an important role in normal and cancer cell. Deregulation of this pathway is reported in various cancers highlighting it as a possible target in cancer. Data suggest that S6K1 is implicated in breast cancer. S6K1 is encoded by the *RPS6KB1* gene localized to the chromosomal region 17q23. Region 17q23 is amplified in several breast cancer cell lines and in about 30% of primary tumours [44], whereas S6K1 is overexpressed in the majority of breast cell lines and breast primary tumours with this amplification [45–48]. In brain and CNS tumours, there is no data regarding the S6 kinases gene expression profile and gene amplification. Using a rich source of publically available cancer microarray data as the Oncomine database, we investigated the gene expression of S6 kinases in different tumours. The overexpression of S6K1 in brain tumours not S6K2 attracted our attention to a distinct role of this homologue in brain tumours. We then surveyed the literature and found no gene expression analysis of S6 kinases in brain tumours has been done. In this study, we interrogated the oncomine database for S6K1 gene expression level and its association patient's clinical outcomes. Furthermore, we investigated the gene expression profile of hypoxia-induced genes and their association with S6K1 gene expression.

Here, we show that S6K1 but not S6K2 is overexpressed in different brain tumours as atypical teratoid tumour, classic medulloblastoma, glioblastoma, and anaplastic oligoastrocytoma compared to normal brain in cancer microarray database; Oncomine (Figure 1). No reports indicated the upregulation of S6K1 in brain tumours at the gene expression level. S6K1 gene is known to be amplified in breast and cervical cancer patients but no reports regarding its amplification in brain tumours have been identified. To test the hypothesis if the overexpression of S6K1 mRNA could be due to its gene amplification, we investigated the oncomine database and found only two records for S6K copy number in brain tumours. No significant difference between S6K DNA copy number in normal and brain tumour was detected in the investigated datasets (data not shown). This excludes the possibility of S6K1 gene amplification and explains that the gene expression upregulation involves upstream gene transcription regulators that bind to S6K1 gene promoter and enhance its expression. We also observed that the overexpression of S6K1 in anaplastic oligoastrocytoma was significantly associated with patients' poor survival status in 3 different analyses, which could attract the attention for S6K1 as a possible marker for prognosis in those brain tumour types. Furthermore, we have observed an association of S6K1 gene overexpression and patients' poor survival in classic medulloblastoma when we analysed Pomeroy et al. study [28] (supplementary Figure  1(a)).

 Due to limited number of studies available on brain tumours and CNS, extending the analysis to other tumours types as glioblastoma retrieved only one study; Murat brain study 2008 [43]. No significant difference was observed between the S6K1 expression levels in the patients' survived after 5 years and patients who died after the same period (Supplementary Figure  2(a)).

Induction of angiogenic factors in tumours is a result for hypoxic induction. The tumours adapt themselves to hypoxic condition by upregulation of HIF1 gene transcription factor which upregulates vascular endothelial growth factor VEGF which is a crucial modulator of new blood vessels formation in tumour. This pathway attracted many studies and is a target for new therapy. The critical role of the hypoxia response network and HIF in cancer has resulted in it being viewed as an ideal target for small molecule intervention (reviewed in [49]). In this study, we investigated the expression profile of hypoxia-induced genes in oncomine cancer microarray database using a literature defined concept. We investigated a list of genes that are known from literature to respond to hypoxia; for example: HIF1, VEGFA, MMP2, SOX9, SOX4, and NEDD9. We have found that all those genes are significantly overexpressed in different brain tumour types compared to normal brain. Furthermore, we showed that the overexpression of S6K1 is associated with the upregulation of hypoxia-induced genes. mTOR/S6K pathway is linked recently to hypoxia mechanism in cancers. Targeting hypoxia and S6K pathway could be a useful tool in brain tumours and better prognosis.

Overall, we report here the induction of S6K1 gene expression in different brain tumours compared to normal brain. This overexpression pattern is associated with patient's poor survival and upregulation of hypoxic-induced genes. These results highlight the possible role of S6K1 in brain tumour progression and as an upstream regulator of hypoxic response. This attracts a further investigation of reported gene combination as a predictor for patients' survival.

## Supplementary Material

Figure 1: (a) S6K1 and survival ( classic medulloblastoma) Promery et al. Nature 2002, (b) S6K1 and survival (glioblastoma) Murat et al. J clin.oncol. 2008.

## Figures and Tables

**Table 1:** Oncomine studies used in this analysis.

Study	Sample type	Patients' numbers	Year of the study	REF
Sun Brain	Normal brain Oligodendroglioma Anaplastic astrocytoma Glioblastoma Diffuse astrocytoma	23 50 19 81 7	2006	[36]

Shai Brain	Glioblastoma Normal white matter7	27 7	2003	[37]

Rickman Brain	Astrocytoma Normal temporal Lobe	45 6	2001	[38]

French Brain	Normal brain Anaplastic oligoastrocytoma Anaplastic oligodendroglioma	6 4 23	2006	[27]

Bredel Brain 2	Glioblastoma Oligodendroglioma Anaplastic oligodendroglioma Anaplastic oligoastrocytoma Normal brain	27 5 3 6 4	2005	[39]

Liang Brain	Glioblastoma Oligoastrocytoma Oligodendroglioma Normal brain Normal cerebellum	29 3 2 2 1	2005	[40]

Gutmann Brain	Pilocytic astrocytoma Normal white matter	8 3	2002	[41]

Pomeroy Brain	Atypical teratoid/rhabdoid tumour Classic medulloblastoma Desmoplastic medulloblastoma Malignant glioma, NOS Primitive neuroectodermal tumour, NOS Normal Brain	5 46 14 10 6 4	2002	[28]

Nutt Brain	Anaplastic oligodendroglioma Glioblastoma	22 28	2003	[29]

Van den Boom Brain	Glioblastoma Mixed glioma Astrocytoma	4 4 8	2003	[42]

Murat Brain	Glioblastoma	80	2008	[43]

Total		622		

**Table 2:** Hypoxia induced genes are overexpressed in human brain tumours.

Gene	Brain tumour type	Upregulation fold/normal	*P* Value	Patients' number	Study/year [REF]
SOX4	Oligodendroglioma	27.195	*P* = 7.16*E* − 22	50	Nutt Brain/2006 [36]
	Anaplastic astrocytoma	22.784	*P* = 1.96*E* − 13	19	
	Glioblastoma	4.149	*P* = 1.06*E* − 21	81	
	Diffuse astrocytoma	9.109	*P* = 0.007	7	
	Glioblastoma	3.084	*P* = 8.51*E* − 8	27	Shai Brain/2003 [37]
	Astrocytoma	6.100	*P* = 1.65*E* − 5	45	Rickman Brain/2001 [38]
	Anaplastic oligoastrocytoma	17.598	*P* = 6.05*E* − 5	4	French Brain/2005 [27]
	Anaplastic oligodendroglioma	2.790	*P* = 4.62*E* − 8	23	
	Glioblastoma	6.140	*P* = 2.76*E* − 9	27	Bredel Brain 2/2005 [39]
	Oligodendroglioma	12.935	*P* = 2.22*E* − 4	5	
	Anaplastic oligodendroglioma	15.078	*P* = 0.002	3	
	Anaplastic oligoastrocytoma	8.939	*P* = 0.006	6	
	Glioblastoma	4.579	*P* = 2.20*E* − 4	29	Liang Brain/2005 [40]
	Oligoastrocytoma	7.482	*P* = 0.009	3	
	Pilocytic astrocytoma	3.445	*P* = 0.008	8	Gutmann Brain/2002 [41]

SOX9	Malignant glioma	4.330	*P* = 9.30*E* − 6	10	Pomeroy Brain/2002 [28]
	Atypical teratoid/rhabdoid tumour	3.668	*P* = 0.007	5	
	Desmoplastic medulloblastoma	2.599	*P* = 0.002	14	
	Anaplastic oligoastrocytoma	4.005	*P* = 0.009	23	French Brain/2005 [27]
	Anaplastic oligodendroglioma	2.731	*P* = 3.03*E* − 4	4	
	Glioblastoma	2.566	*P* = 2.84*E* − 5	27	Shai Brain/2003 [37]
	Anaplastic astrocytoma	2.340	*P* = 2.79*E* − 7	19	Sun Brain/2006 [36]
	Glioblastoma	2.24	*P* = 10*E* − 15	81	

VEGFA	Malignant glioma, NOS	210.500	*P* = 0.005	10	Pomeroy Brain/2002 [28]
	Glioblastoma	9.415	*P* = 1.03*E* − 6	26	Bredel Brain 2/2005 [39]
	Glioblastoma	8.624	*P* = 1.51*E* − 19	81	Sun Brain/2006 [36]
	Anaplastic astrocytoma	2.008	*P* = 0.003	19	
	Pilocytic astrocytoma	7.651	*P* = 0.001	8	Guttman
	Glioblastoma	5.228	*P* = 5.10*E* − 4	30	Liang Brain/2005 [40]
	Glioblastoma	4.998	*P* = 5.28*E* − 8	27	Shai Brain/2003 [37]
	Anaplastic oligoastrocytoma	4.438	*P* = 0.003	23	French Brain/2005 [27]
	Anaplastic oligodendroglioma	3.121	*P* = 7.92*E* − 6	4	

MMP2	Glioblastoma	6.426	*P* = 5.41*E* − 4	27	Bredel Brain 2/2005 [39]
	Glioblastoma	4.537	*P* = 0.003	30	Liang Brain/2005 [40]
	Pilocytic astrocytoma	4.030	*P* = 0.001	8	Gutmann Brain/2002 [41]
	Glioblastoma	3.548	*P* = 7.99*E* − 16	81	Sun Brain/2006 [36]
	Diffuse astrocytoma	3.342	*P* = 0.010	19	
	Anaplastic astrocytoma	2.697	*P* = 1.33*E* − 5	7	
	Anaplastic oligodendroglioma	2.980	*P* = 1.29*E* − 7	23	French Brain/2005 [27]
	Anaplastic oligoastrocytom	2.214	*P* = 0.006	4	
NEDD9	Atypical teratoid/rhabdoid tumour	58.972	*P* = 0.008	5	Pomeroy Brain/2002 [28]
	Malignant glioma, NOS	17.133	*P* = 0.007	10	
	Classic medulloblastoma	7.818	*P* = 3.59*E* − 8	46	
	Desmoplastic medulloblastoma	5.238	*P* = 0.003	14	
	Glioblastoma	2.330	*P* = 3.69*E* − 7	25	Bredel Brain 2/2005 [39]

HIF1A	Oligodendroglioma	3.395	*P* = 0.007	3	Shai Brain/2003 [37]
	Glioblastoma	2.660	*P* = 1.96*E* − 10	27	
	Anaplastic oligoastrocytoma	3.101	*P* = 9.50*E* − 4	4	French Brain/2005 [27]
	Anaplastic oligodendroglioma	2.576	*P* = 4.35*E* − 6	23	
	Glioblastoma	2.954	*P* = 5.21*E* − 9	26	Bredel Brain 2/2005 [39]
	Anaplastic oligodendroglioma	2.404	*P* = 4.78*E* − 4	3	
	Oligodendroglioma	2.167	*P* = 0.003	5	
	Diffuse astrocytoma	2.115	*P* = 6.68*E* − 5	7	Sun Brain/2006 [36]
	Astrocytoma	2.057	*P* = 5.79*E* − 4	45	Rickman Brain/2001 [38]
