# Supplementary material for: Overexpression of S6 Kinase 1 in Brain Tumours Is Associated with Induction of Hypoxia-Responsive Genes and Predicts Patients' Survival
Source: J Oncol. 2012 Apr 5;2012:416927. doi: 10.1155/2012/416927 (PMC3335255; doi:10.1155/2012/416927)
Supplement: Supplementary file 1 — Figure 1: (a) S6K1 and survival ( classic medulloblastoma) Promery et al. Nature 2002, (b) S6K1 and survival (glioblastoma) Murat et al. J clin.oncol. 2008. [file 416927.f1.ppt]

## Slide 1
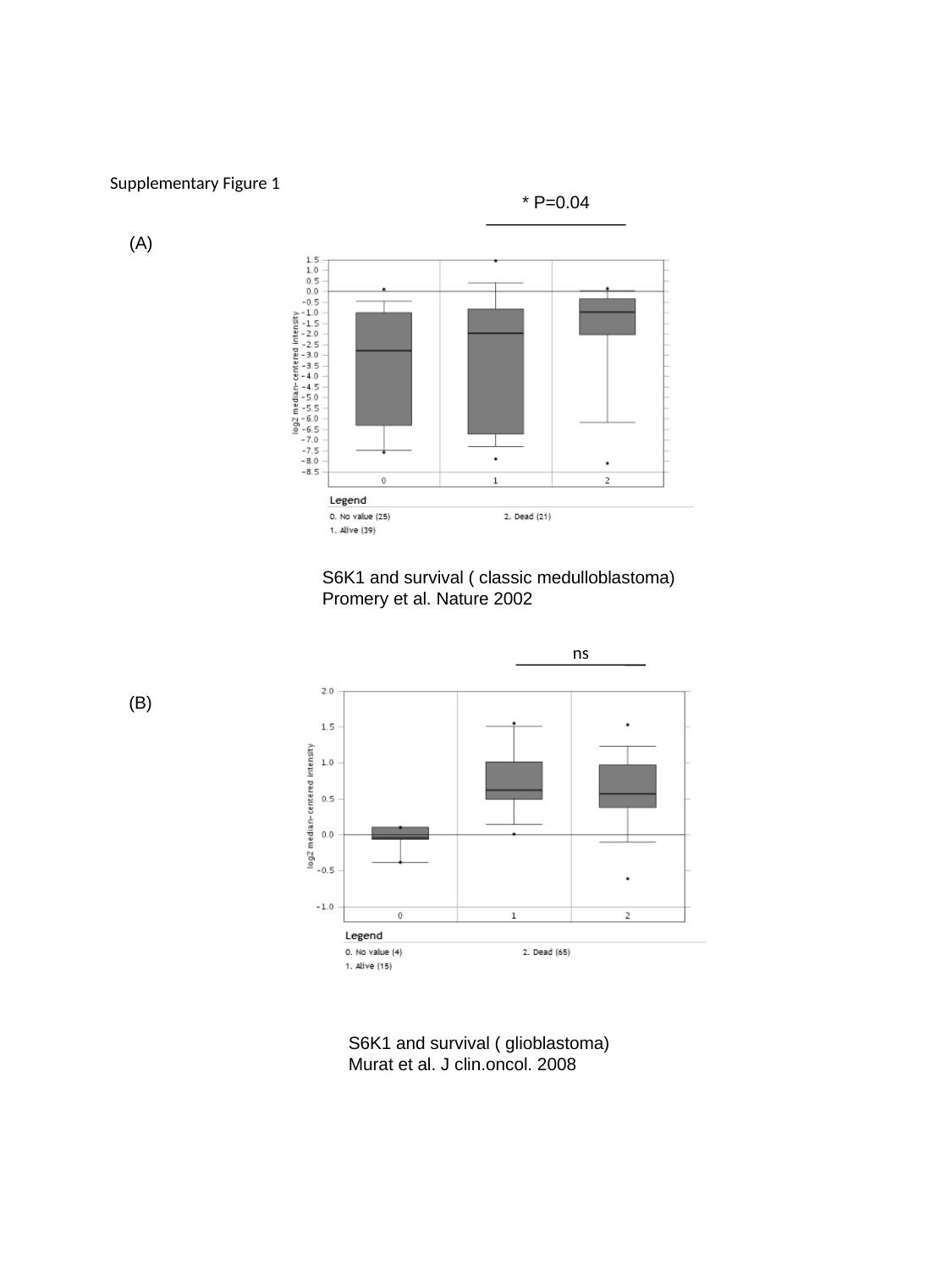

Supplementary Figure 1
* P=0.04
(A)
S6K1 and survival ( classic medulloblastoma)
Promery et al. Nature 2002
ns
(B)
S6K1 and survival ( glioblastoma)
Murat et al. J clin.oncol. 2008
